# Lectures on Geology

**Published:** 1845-04

**Authors:** 


### THE WESTERN JOURNAL.

New Series.—Vol. IV.—No. IV.

LOUISVILLE, APRIL 1, 1845.

### LECTURES ON GEOLOGY.

We were very much interested in a course of lectures on Geology, delivered in this city, during the last month, by Byrem Lawrence, Esq. Mr. Lawrence is a practical geologist, who has explored a large portion of our country for himself, and describes things as they have presented themselves to his own eyes. In the course of the last five years he has traveled 18,000 miles in prosecuting his researches, 8,000 of which he performed on foot. As the reader would infer from this statement, he is an enthusiast in the cause, and with his fine athletic frame in the full vigor of manhood, and a mind capable at once of close, patient observation, and of the most enlarged generalizations, he promises to become a successful and distinguished laborer in this interesting field. What he has observed he describes in a plain, forcible manner, and illustrates his descriptions by a map in which the various formations are so exhibited that his hearers have no difficulty in comprehending the subject.

We have written this notice with the view of calling the attention of our readers to Mr. Lawrence, some of whom, as he is passing through the country, may have an opportunity of hearing him lecture on this most attractive science. Classes ought to be formed for him in every town to which he goes. The information he imparts on points of the most intense interest, great as it is, is the least valuable feature in his lectures: they are calculated beyond anything we have heard, to set men about observing the wonderful things connected with our earth's structure, and to inspire a taste for this noble study.

